# Relationship between Sensory Alterations and Repetitive Behaviours in Children with Autism Spectrum Disorders: A Parents’ Questionnaire Based Study

**DOI:** 10.3390/brainsci11040484

**Published:** 2021-04-11

**Authors:** Anna Fetta, Elisa Carati, Laura Moneti, Veronica Pignataro, Marida Angotti, Maria Chiara Bardasi, Duccio Maria Cordelli, Emilio Franzoni, Antonia Parmeggiani

**Affiliations:** 1 IRCCS Istituto delle Scienze Neurologiche di Bologna, UOC Neuropsichiatria dell’Età Pediatrica, 40139 Bologna, Italy; veronica.pignataro@libero.it (V.P.); maridangotti@hotmail.it (M.A.); ducciomaria.cordelli@unibo.it (D.M.C.); antonia.parmeggiani@unibo.it (A.P.); 2 Dipartimento di Scienze Mediche e Chirurgiche (DIMEC), Università di Bologna, 40137 Bologna, Italy; elisa.carati@studio.unibo.it (E.C.); laura.moneti@studio.unibo.it (L.M.); mariachiara.bardasi@studio.unibo.it (M.C.B.); emilio.franzoni@unibo.it (E.F.)

**Keywords:** ASD, autism, RBS-R, restricted repetitive behaviours, sensory processing disorders, SSP, stereotypy

## Abstract

The relationship between sensory profile and repetitive behaviours in autism spectrum disorder (ASD) has long been known. However, there is no consensus on the type of relationship that exists between them. This monocentric retrospective–prospective observational study aimed (a) to detect a clinical correlation between the severity of repetitive behaviours and the alterations of sensory profile in a sample of 50 children diagnosed with ASD; (b) to evaluate how different patterns of stereotypies and sensory alterations correlate with each other and with the main clinical–instrumental variables in the same sample. We enrolled 29 children in the retrospective phase of the study and 21 in the prospective phase. The Repetitive Behaviour Scale-Revised (RBS-R) and the Short Sensory Profile (SSP) were administered to the caregivers, and clinical–instrumental data were collected. SSP and RBS-R total scores directly correlated with a high significance rate. Among the subscales, the strongest correlations involved “Visual/Auditory Sensitivity”, related to “Stereotyped Behaviour” and “Sameness Behaviour”. “Under-Responsive/Seeks Sensation” related to “Stereotyped Behaviour”. Sex and intellectual disability significantly influenced both the stereotypies and the sensory alterations of the examined population. In conclusion, this study provides new insights into the relationship between sensory alterations and repetitive behaviours in ASD children by using direct medical observation and parent observation.

## 1. Introduction

### 1.1. Restricted, Repetitive Behaviours, and Interests in Autism Spectrum Disorder

Autism spectrum disorder (ASD) is characterised by a deficit in social communication and social interaction, as well as restricted repetitive behaviours and interests. According to the Diagnostic and Statistical Manual of Mental Disorders 5th ed. (DSM-5, APA 2013), restricted repetitive behaviours (RRBs) and interests may manifest themselves in four principal domains: stereotyped and repetitive movements, insistence on sameness, fixed and restricted interests, and anomalies in sensory elaboration (hyper-/hyporeactivity) [1,2].

RRBs and sensory issues compromise the daily functioning of children with ASD by altering their social, cognitive, and motor development [3,4,5].

The DSM5 (APA 2013) [1] definition, as well as previous research [6,7,8,9,10,11,12,13], implies the close connection of sensory alterations to RRBs. However, researchers have yet to reach a consensus on whether RRBs and sensory impairment are related but distinct phenomena [6,7,9] or they are two different aspects of the same phenomenon [14]. Some studies argue that patients with altered sensory elaboration (as are children with ASD) exhibit motor stereotypies when confronting situations with an increased number of sensory inputs, which they perceive as excessively intense and aversive. In such instances, repetitive behaviours would work as a coping mechanism to reduce the excessive hypersensory stimulation and lower the child’s arousal level [11,13,15]. Other studies claim that repetitive behaviours could act as a form of self-stimulation, providing an adequate level of arousal in a condition of hyporesponsiveness [8]. Others combine these two observations and claim that the mechanisms eliciting repetitive behaviours could appear simultaneously, integrated into the same subject, depending on the modality of the presentation of sensory stimuli [16,17].

RRBs are difficult to assess, as they vary and are influenced by many factors. The Repetitive Behaviour Scale-Revised (RBS-R) is a parent-based questionnaire useful to investigate their different facets [18,19].

### 1.2. Sensory Integration, Sensory Processing, and Sensory Modulation in Autism Spectrum Disorder

Alterations in sensory perception are, to date, the most frequently detected anomalies in subjects with Autistic Spectrum Disorder [20] and are documented as early as six months of age in children who will later be diagnosed with ASD [21,22].

Interestingly, parents and siblings of children with ASD report increased levels of altered sensory traits when compared to the general population, supporting a genetic origin of the condition with a wide spectrum of severity [21,23].

Sensory integration theory (SIT), first introduced in 1979 and subsequently further developed, considers sensory processes as the basis of a pyramidal system on which higher functions (cognitive and executive) depend [24,25,26].

Along similar lines, Dunn proposed the existence of a continuum of interactions between neurological threshold, the amount of sensory stimulation required for the nervous system to notice and react to stimuli, and behavioural response. Thus, maladaptive behaviour would arise when this modulation is altered or deficient. This could depend on the sensory threshold (high or low) and the type of response (in agreement or in opposition), resulting in different response patterns [27].

Sensory modulation disorders (SMDs) are impairments in detecting, modulating, interpreting, or responding to sensory stimuli. As a continuum with Dunn’s theories, they are classified into under-responsivity, over-responsivity, and sensation seeking [28,29,30]. They are considered an independent disorder, even though there is a great deal of overlap with the typical symptoms of ASD [31].

Several standardised parent questionnaires have been developed to screen and evaluate SMDs [32,33,34,35]. A recent meta-analysis grouped together the results on their application showing a particular age dependence of manifestations (peak at 6–9 years) and a greater specificity of the under-responsivity pattern in ASDs compared to typical groups [16].

### 1.3. Objectives of the Present Study

The present study aims to analyse, through a clinically oriented approach, considering caregivers’ points of view, RRBs and anomalies in sensory perception and response, their mutual interrelation, and the connection with other clinical aspects in a sample of children with a diagnosis of ASD. It primarily sought to: find a correlation between RRBs and sensory profile, analyse which sensory symptoms were more frequently reported in children with high levels of RRBs and which RRBs were more frequently reported in children with sensory symptoms, and evaluate which sensory symptoms would associate more frequently with which types of RRBs. It also intended to detect correlations between atypical sensory processing, RRBs, and demographic, clinical, and instrumental variables (i.e., sex, age, intellectual disability, sleep disorders, epilepsy, nonspecific Magnetic Resonance Imaging (MRI) brain structural alterations, rehabilitation therapy).

## 2. Materials and Methods

### 2.1. Participants

For our monocentric retrospective–prospective observational study, we recruited 29 idiopathic ASD patients during the retrospective phase and 21 patients during the prospective phase. All patients were under 18 years of age (range: 3–15, median 5) and received an ASD diagnosis based on the International Classification of Diseases and Sanitary Problems in its 10th revision (ICD-10) [30], according to the criteria reported in the DSM-5 (APA 2013) [1]. Moreover, the children were tested with ADOS-2 by one of two certified and experienced psychologists (M.A., V.P.) [36]. Patients over the age of 18 with an uncertain diagnosis or affected by known genetic or metabolic syndromes were excluded.

MRI results were simplified as “normal” or “nonspecific structural alterations” (i.e., nonspecific alterations of the corpus callosum, interhemispheric asymmetries). Patients with pathological brain alterations were excluded.

The clinical characteristics of the study participants were then collected as follows. Epilepsy was defined as at least two unprovoked seizures occurring >24 h apart [37]. Sleep disturbance was considered to be the presence of prolonged sleep onset that requires particular conditions, demanding sleep-onset conditions, a significant delay of sleep onset in the absence of those conditions, and the need for caregiver intervention to return the child to sleep after night waking [38,39]. The presence of intellectual disability or developmental delay was tested using different age-appropriate scales (Bayley-3, WPPSI-III, WISC-IV). Rehabilitation therapies included at least psychomotricity, behavioural therapy, and Augmentative Alternative Communication training.

### 2.2. Measures

Both in the retrospective and the prospective phase of our study, the patients’ caregivers completed the Short Sensory Profile (SSP) [40] and the Repetitive Behaviour Scale-Revised (RBS-R) [41] questionnaires as part of the regular diagnostic process.

The Short Sensory Profile is a caregiver report measure consisting of 38 items, each scored on a 1-point (always) to 5-point (never) Likert scale; thus, lower scores relate to more sensory alterations. The 7 sections of the SSP, corresponding to the principal domains of sensory processing, are Tactile Sensitivity (arousal level in response to tactile stimulation); Taste/Smell Sensitivity (arousal level in response to taste and smell stimulation); Movement Sensitivity (arousal level in response to movement-related stimulation); Under-Responsive/Seeks Sensation (reactivity to sensory stimulation: under-responsivity or sensory seeking); Auditory Filtering (elaboration level of auditive stimulation); Low Energy/Weak (physical energy and tendency to fatigue); Visual/Auditory Sensitivity (arousal level in response to visual and auditory stimulation). Both the subscale scores and the total score are calculated and interpreted on the SSP according to three types of sensory profiles: “Typical presentation”, “Borderline presentation”, and “Atypical presentation”.

The Repetitive Behaviour Scale-Revised is a parent and caregiver-based questionnaire used to screen the frequency and severity of RRBs in children with ASD. It consists of 43 items, each scored on a 0-point (never) to 3-point (always) Likert scale; therefore, higher scores indicate more RRBs. The 43 items in the RBS-R are assessed across 6 subscales, each corresponding to one of the principal typologies of RRBs: Stereotyped Behaviour (repetitive actions or movements without an apparent goal), Self-Injurious Behaviour (repetitive actions or movements causing bruises, redness, or other types of lesions), Compulsive Behaviour (repetitive behaviours accomplished following specific rules), Ritualistic Behaviour (everyday life actions that must always be accomplished in the same way), Sameness Behaviour (resistance to change; insistence for things to remain as they are) and Restricted Interests (limited interests or activities range).

### 2.3. Data Collection and Analysis

Demographic information (sex, age), ADOS level of autism-related symptoms (low, moderate or high), MRI results), clinical variables (epilepsy, sleep problems, intellectual disability, rehabilitation therapy), and SSP and RBS-R scores have been collected in anonymised form and recorded in an Excel database.

In the descriptive analysis, continuous variables were presented through mean and standard deviation or median and interquartile range, categorical variables through absolute numbers and percentages. The distributional normality of the variables was assessed by means of the Shapiro–Wilk test.

The relationships between the SSP and RBS-R total scores and subscales were evaluated with the Spearman Rho coefficient. Due to the large number of correlations, the statistical significance threshold was placed at *p* < 0.001. Secondly, nonparametric regression was used to assess the dependence of the RBS-R total score on the SSP total score.

The nonparametric Wilcoxon test was used to compare SSP and RBS-R scores according to demographic (sex, age groups 2–3/4–6/>6), clinical (intellectual disability, sleep disorders, epilepsy, rehabilitation therapy), and instrumental (nonspecific MRI alterations) variables. The classic statistical significance threshold *p* < 0.05 was set. The analyses were performed using Stata/SE version 14.2 software.

## 3. Results

The participants demographic and clinical-instrumental variables are listed in Table 1.

### 3.1. Sensory Profile, Repetitive Behaviours, and Their Mutual Correlation

The SSP and RBS-R subscale scores are reported in Table 2 and Table 3.

The correlation study between the SSP and RBS total scores was highly significant (Rho = −0.80 (*p* < 0.001)).

**Figure 1:**
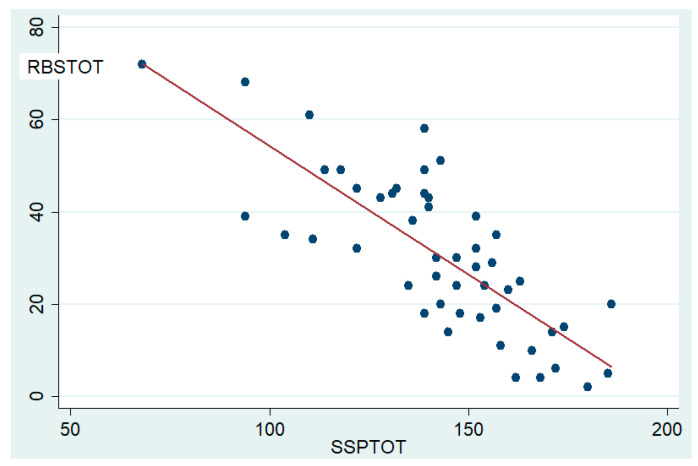
Correlation between sensory profile and repetitive behaviours. Legend: The scatter plot shows the linear correlation, with a low dispersion grade, between SSP total scores (SSP-TOT) and RBS-R total scores (RBS-TOT).

A nonparametric regression analysis was also carried out (F(1,48) = 77.96 *R*^2^ = 0.611; *p* < 0.001), showing that more than 61% of the variability of RBS-TOT is explained by the variable SSP-TOT (Figure 1).

Correlation analysis between the SSP and the RBS-R subscales showed numerous significant correlations (Table 4).

### 3.2. Influence of Demographic and Clinical Variables on Repetitive Behaviour and Sensory Alterations

**Figure 2:**
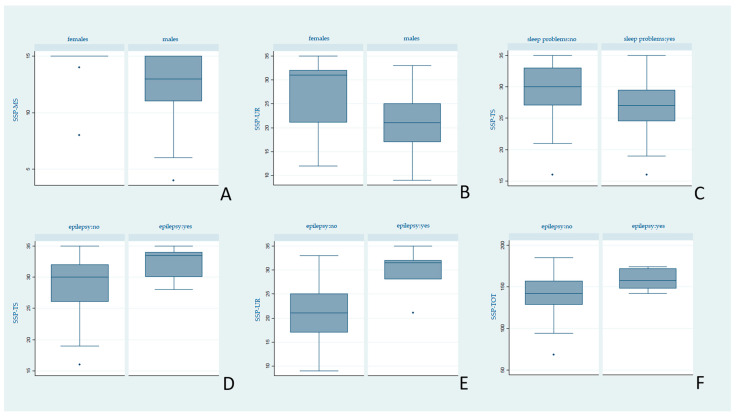
Differences in SSP median scores by clinical variables. Legend: Higher scores indicate less anomalies. (**A**,**B**) Males showed higher symptoms in Movement Sensitivity (SSP-MS) and Under-Responsive/Seeks Sensations (SSP-UR) compared with females, (*p* = 0.01, *p* = 0.03 respectively). (**C**) Subjects with sleep problems presented higher anomalies in Tactile Sensitivity (SSP-TS) than subjects without sleep problems (*p* = 0.04). (**D**–**F**) Subjects with epilepsy displayed less anomalies in Tactile Sensitivity (SSP-TS) (*p* = 0.02), Under-Responsive/Seeks Sensations (SSP-UR) (*p* = 0.008) and SSP total score (SSP-TOT) (*p* = 0.04) than subjects without epilepsy.

**Figure 3:**
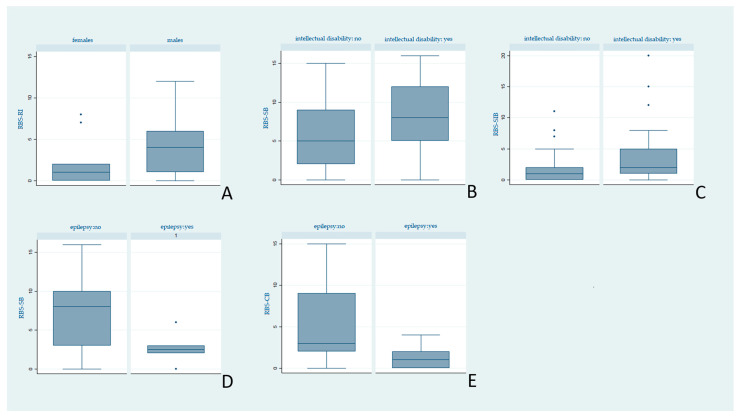
Differences in RBS-R median scores by clinical variables. Legend: Higher scores indicate more impairment. (**A**) Males showed more impairment in Restricted Interest (RBS-RI) than females (*p* = 0.02). (**B**,**C**) Patients with intellectual disability displayed higher impairment in Stereotypic Behaviour (RBS-SB) and Self-Injurious Behaviour (RBS-SIB) than patients with normal QI (*p* = 0.03, *p* = 0.02, respectively). (**D**,**E**) Subjects with epilepsy showed less impairment in Stereotypic Behaviour (RBS-SB) and Compulsive Behaviour (RBS-CB) than patients without epilepsy (*p* = 0.02, *p* = 0.02, respectively).

As a secondary aim of our study, both the SSP and the RBS-R scores were analysed according to demographic and clinical variables. The statistical significance threshold was placed at *p* < 0.05. Statistically significant results are reported in Figure 2 and Figure 3.

We did not find any significant differences depending on age, rehabilitation therapy, or the presence of nonspecific alterations to the encephalic MRI scan among the SSP and RBS-R total and subscale scores. The full statistical analysis can be found in the supplements (Tables S1 and S2).

## 4. Discussion

The primary aim of the study was to evaluate the strength and the type of correlation between sensory profile alterations and the severity of RRBs in ASD patients.

As expected, similarly to previous scientific research [9,13,17,42], a highly significant correlation between sensory profile alterations (SSP total score) and repetitive behaviours (RBS-R total score) emerged.

Regression analyses confirmed this and showed a strong influence of SSP-tot on RBS-tot. With the limitations of a parent-based study and the presence of some overlapping questions between the two questionnaires, this result fits well with the theory that RRBs are a manifestation of sensory modulation disorder in these patients [6,7,8,9,10,11,12,13].

In considering the correlations between the subscales in our sample, some data stood out. Auditory and visual sensory alterations (SSP-VA) and tactile sensitivity (SSP-TS) were correlated with different items on the RBS-R scale. The relationship with stereotypes and repetitive behaviours (RBS-RB), sameness (RSS-SB) and compulsive behaviours (RBS-CB) has long been known [13,42,43,44]. The theory of arousal considers them as a homeostatic mechanism of compensation for the constitutive hyperresponsiveness of the ascending reticular formation; stereotypies would serve as substitutive movements aimed at blocking the sensory input in the case of hyperarousal. Visual and auditory hypersensitivity would lead to acts of avoidance, annoyance, and escape that are manifested through stereotypies [8,11]. The presence of certain stereotypies such as “bringing one’s hands to the ears” and the “angular gaze/out of the corner of the eye”, almost exclusively in subjects with ASD [45,46], reinforces this hypothesis. Baranek et al. propose that ASD children with a particular aversion to tactile stimulation could develop behaviours characterised by inflexibility, rigidity, and search for immutability in order to make their interaction with the environment repetitive and predictable [47].

On the other hand, compulsive behaviours associated with immutability (sameness) also characterise other disorders, including obsessive–compulsive disorder (OCD), which can occur in comorbidity with ASD [48]. The sensory hyperreactivity experienced by ASD patients would generate a condition of anxiety relief, like in OCD, through compulsive, repetitive behaviours [7,49]. Similarly, individuals with altered reactivity to sensory stimuli may show alterations in the attentional focus, which could be particularly fixed and difficult to shift or could be abnormally directed to single parts of an object. This would lead to a necessary repetitiveness and immutability and a strong resistance to change [50,51].

The subscale “Under-Responsiveness/Searching for Sensations”, as the name suggests, includes both hyporesponsiveness and the search for stimuli based on the assumption that one is the manifestation of the other. As reported, Under-Responsiveness seems to be the most typical manifestation of SMDs in ADS patients [16]. In our population, it correlated with both stereotypical and repeated behaviours (RBS-SB and RBS-RB), complying with the theory of arousal: stereotypies would result as alternative sources of sensory stimulation in the case of hypostimulation or sources of stimulus in conditions of hypoarousal. The items related to “Search for Sensations”, i.e., “Enjoys/Produces Noise”, agitation and continuous movement and continuous manipulation of objects, seem to fit this description particularly well [17,52]. The repetition of stereotyped behaviour would derive from the positive sensory feedback produced by the behaviour itself [53,54].

The correlation of the same RBS-R items with both hypo- and hyperresponsiveness SSP items fits in with Dunn’s theories on sensory modulation [27], and it is likely, as some authors suggest, that both modes could be present in the same individual [17,52].

As the second endpoint, we aimed to find significant correlations between RRBs, sensory profile alterations, and demographical, clinical, and instrumental variables. The SSP and RBS-R scores of our sample are similar to those reported in the literature in ASD populations [18,35]. However, contrary to previous reports [16], we found no severity difference between age groups. Males obtained worse scores than females both in the SSP and RBS-R subscales, confirming the known peculiar biological and etiopathogenetic gender specificities. However, it cannot be excluded that the lower presence of RRBs in females is a consequence of a gender bias, intrinsic in most of the evaluation questionnaires (RBS-R included). They are very often oriented towards detecting, with greater sensitivity, typically pathological male characteristics, underestimating female ones [55,56,57].

Consistent with previous evidence [3,46], intellectual disability correlates with increased stereotypes and self-harming behaviour (SSP- SB and SSP-SI). Although the prevalence of self-injurious acts in ASD is estimated to be around 40–50% [58,59], they are not specific to ASD [55,60] but are more typical of patients with severe symptoms and severe intellectual deficits [61,62]. Recent evidence of an association between intellectual disability, repetitive-narrow behaviours, and hyperactivity and impulsivity [63,64] has led some authors to suggest that deficits in executive functions (particularly the impairment of the inhibitory function) would affect the severity and frequency of self-injurious acts. The subject may be less able, less inclined to stop a stereotyped/self-injurious behaviour once the behavioural pattern has started [62,65].

Another remarkable result is the correlation between sleep disorders and tactile anomalies (SSP-TS). Our data support what has recently been reported by Tzischinsky et al. [66], with whom we agree that the presence of hypersensitivity, especially tactile, would be responsible for alterations in falling asleep and maintaining the sleep state. The simultaneous presence of anxiety and hyperarousal could augment the relationship between the two. More in-depth studies in this area could offer new insights for the treatment of sleep disorders, frequently reported in ASD children (25% of patients considered by us) and strongly impacting the quality of life of young patients.

Interestingly, although a large part of our sample (36 patients out of 48) had already undergone a rehabilitative treatment at the time of the evaluation questionnaires, no differences emerged between this group and the group of patients who were not receiving treatment, in any of the scales of the SSP and RBS-R questionnaires. These data would seem to highlight the absence of a significant influence of the rehabilitative treatment on the trend of sensory alterations and RRBs. However, it should be considered that the observational (and partly retrospective) nature of our study did not allow us to consider individual types of rehabilitation therapy and their timing. Moreover, treatments targeted to improve these manifestations (Sensory Integration Therapy, PRT, and sensory extinction therapy), whose effectiveness is however not unequivocal [20,53,67,68], are not always included in clinical practice and/or started early.

A final point of reflection is the highest number of significant correlations of epilepsy with both the SSP and RBS-R scales. Surprisingly, epileptic patients seem to have fewer sensory alterations and RRBs compared to patients without epilepsy. Both epilepsy and altered sensory profile are manifestations of a neurodevelopmental disorder that affects normal neuronal functioning [69]. Hence, we expected a lowered sensory threshold and a worse sensory profile in epileptic patients, as reported in previous studies [70]. Similarly, although not much data are available on stereotypies in epileptic and nonepileptic ASD patients, the characteristic hyperexcitability would suggest a greater severity of stereotypes in the former [71,72].

These data are difficult to explain and are not supported by the literature. Several factors could have affected this result, including the small sample size and the possible underestimation of sensory disturbances by parents coping with the presence of epileptic seizures.

Interestingly, valproic acid, one of the main antiepileptic drugs used also in our population, has been proved to have a positive effect on irritability in patients with ASD using the Aberrant Behaviour Checklist (which includes stereotypes among the various items [73,74]). We therefore speculate that the lower severity of RRBs we found in epileptic patients could be an effect of antiepileptic treatment. Further studies with a better characterisation of the epileptic phenotype and pharmacological profile may clarify this aspect in the future.

### Limitations

Our study presents some limitations. First of all, as in the case of all self-report questionnaires, reports may have been influenced by the level of perception of the problem by caregivers. Moreover, because this is an observational study on data normally collected in clinical practice, we have no data regarding the characteristics of the caregiver who completed the questionnaire.

Furthermore, children with ASD may present exhibit different sensory manifestations in different contexts (for example home and school), which have not been considered. Further targeted studies with questionnaires addressed to teachers and educators would be useful to investigate this aspect.

## 5. Conclusions

Our study examines the correlations between the sensory profile and repetitive behaviours in ASD patients from a clinical point of view, considering caregivers’ opinions. The main novelty introduced by our research is the simultaneous use and comparison of SSP and RBS-R questionnaires, which might allow, thanks to the data collected from the caregivers, to better understand and relate the clinical features of the heterogeneous sensory and behavioural manifestations characterising ASD. From a clinical perspective, these observations may be useful for practitioners to investigate the various types of stereotypies also through the use of simple questionnaires in order to use them as symptoms of underlying sensory anomalies and provide guidance in modulating rehabilitation therapy. In the future, recognising the identification of increasingly defined relationships will enable to set up increasingly effective treatment strategies to modify the impact that these alterations, and their behavioural manifestations, have on patients’ lives.

## Figures and Tables

**Table 1:** Demographic and clinical–instrumental variables.

Variable (Sample Size)		*N* (%)
Gender (50)	Female	11 (22%)
	Male	39 (78%)
ADOS-2: level of autism-related symptoms (50)	Low	18 (36%)
	Moderate	22 (44%)
	High	10 (20%)
MRI (40)	Normal	36 (90%)
	Nonspecific structural alterations	4 (10%)
Epilepsy (49)	Yes	43 (88%)
	No	6 (12%)
Intellectual Disability (49)	Yes	31 (63%)
	No	18 (37%)
Sleep disorders (48)	Yes	36 (75%)
	No	12 (25%)
Rehabilitation therapy (48)	Yes	12 (25%)
	No	36 (75%)

**Table 2:** Short Sensory Profile (SSP) mean subscales and total scores.

SSP Subscales	Mean (SD)
Tactile Sensitivity	29.0 (4.7)
Taste/Smell Sensitivity	14.0 (5.0)
Movement Sensitivity	13.0 (2.6)
Under-Responsive/Seek Sensation	22.6 (6.6)
Auditory Filtering	20.0 (5.5)
Low Energy/Weak	25.6 (5.6)
Visual/Auditory Sensitivity	18.7 (4.4)
Total	143 (24.0)

**Table 3:** Repetitive Behaviour Scale-Revised (RBS-R) mean subscales and total scores.

RBS-R Subscales	Mean (SD)
Stereotyped Behaviour	6.4 (4.4)
Self-injurious Behaviour	2.9 (4.0)
Compulsive Behaviour	4.7 (4.4)
Ritualistic Behaviour	4.6 (3.3)
Sameness Behaviour	8.0 (5.7)
Restricted Interests	3.8 (3.3)
Total	30.3 (17.0)

**Table 4:** Spearman correlation analysis between SSP and RBS-R subscales and total scores.

	RBS−SB	RBS−SIB	RBS−CB	RBS−RB	RBS−SAB	RBS−RI	RBS−TOT
SSP−TS	−0.39	−0.37	−0.50 *	−0.34	−0.53 *	−0.54 *	−0.58 *
SSP−TSS	−0.27	−0.17	−0.32	−0.39	−0.26	−0.39	−0.39
SSP−MS	0.02	−0.19	−0.04	−0.28	−0.34	−0.38	−0.28
SSP−UR	−0.64 *	−0.39	−0.46	−0.21	−0.41	−0.50 *	−0.61 *
SSP−AF	−0.37	−0.38	−0.35	−0.24	−0.54 *	−0.46	−0.54 *
SSP−LE	−0.10	−0.01	−0.11	−0.24	−0.37	−0.43	−0.27
SSP−VA	−0.61 *	−0.44	−0.53 *	−0.35	−0.65 *	−0.47	−0.79 *
SSP−TOT	−0.59 *	−0.40	−0.59 *	−0.44	−0.70 *	−0.70 *	−0.80 *

Caption: The table shows the correlations between RBS and SSP through the Rho Spearman rank correlation coefficient. Rho 0.90–0.71 = strong correlation grade. Rho 0.70–0.51 = good correlation grade. Rho 0.50–0.41 = moderate/mild correlation grade. Rho 0.40–0.31 = low correlation grade. Rho < 0.30 absent correlation. * = *p* < 0.001. RBS-SB: Stereotypic Behaviour. RBS-SIB: Self-injurious Behaviour. RBS-CB: Compulsive Behaviour. RBS-RB: Ritualistic Behaviour. RBS-SAB: Sameness Behaviour. RBS-RI: Restricted Interest. RBS-TOT: RBS-R total score. SSP-TS: Tactile Sensitivity. SSP-TSS: Taste/Smell Sensitivity. SSP-MS: Movement Sensitivity. SSP-UR: Under-Responsive/Seeks Sensations. SSP-AF: Auditory Filtering. SSP-LE: Low Energy/Weak. SSP-VAS: Visual/Auditory Sensitivity. SSP-TOT: SSP total score.

## Data Availability

The data presented in this study are available on request from the corresponding author. The data are not publicly because dissemination has not been explicitly foreseen by the local ethics committee.

## Supplementary Materials

The following are available online at https://www.mdpi.com/article/10.3390/brainsci11040484/s1, Table S1: Wilcoxon comparison analysis between SSP subscales median scores and clinical-instrumental variables. Table S2: Wilcoxon comparison analysis between RBS-R subscales median scores and clinical-instrumental variables.
