# Supplementary material for: Relationship between Sensory Alterations and Repetitive Behaviours in Children with Autism Spectrum Disorders: A Parents’ Questionnaire Based Study
Source: Brain Sci. 2021 Apr 11;11(4):484. doi: 10.3390/brainsci11040484 (PMC8069400; doi:10.3390/brainsci11040484)
Supplement: Supplementary file 1 [file brainsci-11-00484-s001.pdf]

Table S1. Wilcoxon comparison analysis between SSP subscales median scores and clinical-instrumental variables.

**Variable** (sample size)	SSP-TS	SSP-TSS	SSP-MS	SSP-UR	SSP-AF	SSP-LE	SSP-VAS	SSP-TOT
**Age** (50)								
2-3	30 (29.5-31.5)	14 (10-18)	15 (13-15)	17.5 (21-23.5)	21 (17-22.5)	28 (21.5-30)	19 (17-21.5)	141 (137.5-151)
4-6	27.5 (25.5-32)	14 (8.5-18.5)	14 (11.5-15)	20.5 (17.5-23.5)	21.5 (16-22.5)	29.5 (25.5-30)	19 (17-22)	143.5 (122-158.5)
>6	31 (26-34)	16 (12-20)	14 (12-15)	26 (21-32)	22 (17-26)	27 (21-29)	19 (11-23)	152 (139-163)
p	.4	.46	.27	.08	.78	.17	.98	.74
**Gender** (50)								
Female	33 (28-34)	14 (10-20)	15 (15-15)	31 (21-32)	21 (16-24)	29 (27-30)	20 (16-24)	152 (139-172)
Male	30 (26-32)	15 (10-19)	13 (11-15)	21 (17-25)	22 (16-24)	28 (21-30)	19 (15-22)	143 (128-157)
p	.07	.93	**.01***	**.03***	.74	.37	.46	.11
**Brain MRI: non-specifical structural alterations** (40)								
NO	30 (26.5-32)	15 (12-19)	14 (12-15)	21 (17.5-27)	21.5 (17-23)	28.5 (22-30)	19 (14.5-21)	146 (133-157.5)
YES	28.5 (24-30)	11.5 (7-17)	11 (8.5-13)	19.5 (17.5-21.5)	16 (16-19)	23.5 (21-26)	17.5 (16-20)	127 (116.5-140)
p	.34	.35	.14	.5	.21	.18	.86	.14
**Epilepsy** (49)								
NO	30 (26-32)	15 (10-18)	14 (12-15)	21 (17-25)	21 (16-24)	28 (21-30)	19 (16-22)	142 (128-157)
YES	33.5 (30-34)	19 (14-20)	13 (11-15)	31.5 (28-32)	22 (22-22)	27 (23-29)	20.5 (15-24)	157.5 (148-172)
p	**.02***	.1	.77	**.008***	.29	.42	.67	**.04***
**Intellectual Disability** (49)								
NO	29 (26-32)	15 (9-19)	14 (12-15)	22 (17-29)	21 (16-24)	28 (21-30)	19 (18-22)	147 (128-158)
YES	30 (27-33)	15 (12-19)	14.5 (12-15)	21 (18-24)	22 (17-23)	29 (24-30)	16.5 (13-22)	142.5 (139-152)
p	.5	.75	.68	.37	.8	.32	.1	.72
**Sleep Disorders** (48)								
NO	30 (27-33)	15.5 (12-19)	14 (12-15)	21 (18-26.5)	22 (16-23)	28 (23.5-30)	19 (16.5-22)	147 (135.5-158)
YES	27 (24.5-29.5)	12 (5.5-15)	13 (10.5-15)	20.5 (15-27)	21 (13.5-23)	22.5 (16.5-30)	17.5 (11.5-19.5)	139 (106.5-148)
p	**.04***	.06	.38	.66	.47	.12	.08	.09
**Rehabilitation therapy** (48)								
NO	30.5 (26-31.5)	12.5 (7.5-17.5)	14 (12-15)	21 (16-26.5)	22 (12.5-24)	27.5 (20-30)	22 (16.5-22.5)	139.5 (133-156.5)
YES	29.5 (26.5-33)	15 (12-19)	14 (12-15)	21 (18-27)	21 (16.5-22)	28 (21.5-30)	19 (15.5-20)	144 (130-157.5)
p	.9	.26	.77	.81	.78	.58	.22	.72

Caption: * = $p<0.05$; SSP-TS: Tactile Sensitivity; SSP-TSS: Taste/Smell Sensitivity; SSP-MS: Movement Sensitivity; SSP-UR: Under responsive/Seeks sensations; SSP-AF: Auditory Filtering; SSP-LE: Low Energy/Weak; SSP-VAS: Visual/Auditory Sensitivity; SSP-TOT: SSP total score.

Table S2. Wilcoxon comparison analysis between RBS-R subscales median scores and clinical-instrumental variables.

Variable (sample size)	RBS-SB	RBS-SIB	RBS-CB	RBS-RB	RBS-SAB	RBS-RI	RBS-TOT
Age (50)							
2-3	7 (5-8.5)	2 (0.5-2)	3 (2-6.5)	2.5 (0-5.5)	6 (3-9.5)	2.5 (0.5-4.5)	22 (16-39.5)
4-6	6 (2.5-10)	2 (0-4.5)	2.5 (1-9.5)	5 (2-7)	7.5 (2.5-13)	4 (2-6)	34.5 (20.5-43.5)
>6	3 (1-10)	1 (0-3)	4 (2-7)	6 (3-9)	7 (5-14)	2 (1-6)	29 (17-49)
P	.85	.61	.92	.2	.77	.31	.87
Gender (50)							
F	5 (1-8)	1 (0-2)	2 (0-6)	3 (2-6)	6 (3-8)	1 (0-2)	20 (14-30)
M	7 (3-11)	2 (0-4)	3 (2-10)	6 (1-7)	8 (4-13)	4 (1-6)	34 (19-44)
P	.15	.17	.25	.81	.2	**.02***	.06
MRI non-specifical alterations (40)							
NO	8 (3-11)	2 (1-4.5)	4 (2-9.5)	4 (2-6.5)	7.5 (4-11.5)	2.5 (1-6)	31 (20-44)
YES	5.5 (2-9)	2 (0-4.5)	1.5 (0.5-4)	7 (4-9)	14 (10-15.5)	5.5 (3-9.5)	39.5 (26-45)
P	.44	.65	.11	.26	.08	.15	.56
Epilepsy (49)							
NO	8 (3-10)	1 (0-3)	3 (2-9)	5 (1-7)	8 (3-13)	3 (1-6)	32 (18-44)
YES	2.5 (2-3)	3 (0-8)	1 (0-2)	2 (2-4)	5.5 (4-8)	1.5 (0-2)	21.5 (15-26)
P	**.02***	.54	**.02***	.28	.31	.15	.09
Intellectual Disability (49)							
NO	5 (2-9)	1 (0-2)	2 (1-5)	4 (2-7)	7 (2-11)	4 (1-6)	26 (11-43)
YES	8 (5-12)	2 (1-5)	6.5 (2-9)	4 (2-6)	8.5 (4-14)	2 (1-5)	31(23-49)
P	**.03***	**.02***	.07	.86	.44	.28	.16
Sleep Disorders (48)							
NO	5 (2-10)	1.5 (0-3.5)	3 (1.5-7)	3.5 (1-6)	7 (4-10.5)	2 (1-5)	28.5 (16-41)
YES	8 (5.5-10)	1.5 (0-6.5)	5.5 (2-10)	5.5 (3.5-8.5)	10.5 (5.5-15)	6 (2-10)	40 (25-53.5)
P	.21	.82	.17	.11	.12	.07	.06
Rehabilitation therapy (48)							
NO	6.5 (1.5-11.5)	1.5 (0.5-4)	4 (1.5-8.5)	3.5 (1-7)	6.5 (2-11.5)	2 (1-5)	26.5 (18-43.5)
YES	6 (3-9.5)	1.5 (0-4)	3 (2-7.5)	4.5 (2-6.5)	8 (4-12.5)	3.5 (1-6)	31(18.5-43.5)
P	.94	.73	.67	.70	.52	.36	.67

Caption: * = $p<0.05$; RBS-SB: Stereotypic Behaviour; RBS-SIB: Self-injurious Behaviour; RBS-CB: Compulsive Behaviour; RBS-RB: Ritualistic Behaviour; RBS-SAB: Sameness Behaviour; RBS-RI: Restricted Interest; RBS-TOT: RBS-R total score.
